# Endogenous Human Proteins Interfering with Amyloid Formation

**DOI:** 10.3390/biom12030446

**Published:** 2022-03-14

**Authors:** Anna L. Gharibyan, Sanduni Wasana Jayaweera, Manuela Lehmann, Intissar Anan, Anders Olofsson

**Affiliations:** 1 Department of Clinical Microbiology, Umeå University, 901 87 Umeå, Sweden; sanduni.jayakodi@umu.se; 2 Department of Public Health and Clinical Medicine, Umeå University, 901 87 Umeå, Sweden; manuela.lehmann@umu.se (M.L.); intissar.anan@umu.se (I.A.)

**Keywords:** amyloid inhibition, endogenous proteins, transthyretin, apolipoprotein E, clusterin, BRICHOS, amyloid-beta, IAPP, alpha-synuclein

## Abstract

Amyloid formation is a pathological process associated with a wide range of degenerative disorders, including Alzheimer’s disease, Parkinson’s disease, and diabetes mellitus type 2. During disease progression, abnormal accumulation and deposition of proteinaceous material are accompanied by tissue degradation, inflammation, and dysfunction. Agents that can interfere with the process of amyloid formation or target already formed amyloid assemblies are consequently of therapeutic interest. In this context, a few endogenous proteins have been associated with an anti-amyloidogenic activity. Here, we review the properties of transthyretin, apolipoprotein E, clusterin, and BRICHOS protein domain which all effectively interfere with amyloid in vitro, as well as displaying a clinical impact in humans or animal models. Their involvement in the amyloid formation process is discussed, which may aid and inspire new strategies for therapeutic interventions.

## 1. Introduction

Amyloidosis refers to a variety of increasingly common human diseases, including Alzheimer’s disease (AD), Parkinson’s disease (PD), and diabetes mellitus type 2 (T2D). Amyloids are composed of insoluble proteinaceous material with a β-sheet rich fibrillar morphology that can reach several microns in length and are typically between 5–10 nm in diameter. They can arise from different types of proteins through a multistep process, known as amyloidogenesis. Up to date, more than 30 different proteins and peptides have been identified to form amyloid structures in vivo [1]. These polypeptides are encoded by separate genes without sequence homologies or functional similarities. However, in their amyloid form, they all acquire similar morphologies including a cross-β sheet structure and similar tinctorial properties upon staining with thioflavin or Congo red [2].

**Figure 1:**
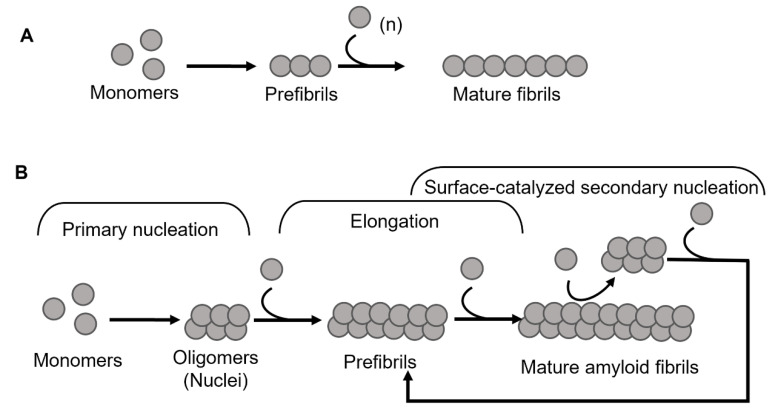
Schematic models of amyloid fibril formation. (**A**) Linear polymerization, where partially unfolded or misfolded monomers assemble into short prefibrillar structures and by sequential addition of monomers elongate into mature fibrils. (**B**) Nucleation-dependent amyloid formation, where the monomers form soluble oligomeric nucleus during the lag phase, then assemble into larger prefibrillar structures followed by elongation and subsequently a saturation phase where mature fibrils are formed. Regarding some amyloid proteins, mature fibrils can serve as a template for the formation of new nuclei, resulting in a rate-enhancing process known as surface catalyzed secondary nucleation.

Amyloid formation in general follows a polymerization process that can be described by two basic models, being either linear (also known as isodesmic self-assembly) or nucleation-dependent polymerization (Figure 1) [3]. The linear polymerization process is initiated by a monomeric subunit followed by the continuous addition of monomers. The successive incorporation of monomers to the growing polymers is energetically favorable without the need for a preformed nucleus (Figure 1A). The formation of amyloid via the linear path results in a hyperbolic curvature initiated at time zero. In contrast to this reaction, the nucleation-dependent path of polymerization has a slow initial step in the reaction kinetics, where several molecules assemble to form an oligomeric nucleus before elongation is initiated (Figure 1B). This period is defined as a lag phase. Consequently, the nucleus serves as a template for the addition of monomeric molecules. Regarding the formation of an oligomeric species the energy barrier is significantly higher than the template-dependent incorporation of monomers onto a fibrillar end, and once enough initiation sites have been formed, the process of elongation dominates the reaction. The rapid process of elongation is followed by a saturation phase where the reaction reaches a steady state when most monomers in the system have been converted into fibrillar structures and where the association and dissociation rates are equal.

For most of the polypeptides, amyloid formation occurs via a nucleation-dependent mechanism [4]. However, there is evidence of linear downstream polymerization leading to amyloid formation, as it is described for transthyretin (TTR) [5]. In the case of some amyloids, such as Aβ [6], α-synuclein [7], and IAPP [8] the surface of mature fibrils can serve as sites that catalyze the formation of new nuclei and hence further enhance the rate of the reaction. This mechanism is called surface-catalyzed secondary nucleation (SCSN) [6,7,8,9], (see Figure 1B).

Amyloid formation in vivo frequently leads to pathological changes in the affected organs, including tissue and cell degeneration and inflammation. Although the cell and tissue damages vary between different amyloid forms, the associated pathological effects are often linked to cytotoxic properties of amyloid structures. This has been observed both in vitro, on various cell lines, and in vivo on animal models [1,10,11]. There is also a consensus that prefibrillar structures, especially early soluble oligomers, induce higher toxicity than the mature fibrils [12,13,14]. The relative toxicity, however, seems to vary significantly between different amyloids and is likely a function of both their properties as well as their site of deposition. For example, the total load of the Aβ-amyloid deposits in the brain of an AD patient corresponds to only a few milligrams [15], nevertheless causing neuroinflammation and massive degeneration of neuronal cells. In contrast, lysozyme-amyloidosis may generate a total load of amyloid corresponding to several kilograms [16], but the pathological mechanism is rather a rupture of the organs due to a high load of aggregates than an acute cytotoxic effect.

Experimental data on animal and cell models suggest that inhibition of amyloid formation is essential for preventing or suppressing the progression of the pathological process [17,18].

In the quest to prevent amyloid formation, several different strategies have been explored. Among these, a range of small molecules of both synthetic [19,20,21] and natural origin [22,23] have been evaluated. However, the main problem with most of these compounds is the translation of laboratory observations into the clinic and application in vivo, where problems of bioavailability and safety hamper their use. Another approach is the development of specific anti-amyloid antibodies for immunotherapy, so far mostly explored against Aβ in AD [24,25,26] and α-synuclein in PD treatment [27,28]. However, most of these antibodies failed clinical trials due to the lack of efficiency in the improvement of pathological conditions and safety problems (reviewed in [27,29]).

A small number of endogenous proteins that display amyloid-interfering properties have potential therapeutic value, as they can serve as a more natural way of preventing amyloid formation by promoting self-regulation of protein homeostasis. Among such proteins are molecular chaperons and several proteins with diverse physiological functions, including transport. In the early nineties, it was discovered that in extracellular fluids such as cerebrospinal fluid (CSF) and serum, the highly amyloidogenic amyloid-β peptide (Aβ) forms stable complexes with some proteins and does not aggregate into amyloid [30,31,32]. These proteins were also found colocalized with senile plaques, suggesting their involvement in regulation and maintenance of amyloid-prone polypeptides, and that failure in this process potentially could lead to pathological amyloid formation and deposition.

In this review, we summarize the information accumulated in the literature regarding some of these proteins, which have been intensively studied in recent years, namely, TTR [33,34,35,36,37], Apolipoprotein E (ApoE) [31,38,39,40,41,42], clusterin (also known as Apolipoprotein J) [43,44], and the more recently identified BRICHOS protein domain [45,46,47,48]. Understanding their role in pathology and their mechanisms of action can help in the development of new therapeutic strategies. Therefore, we will give an overview of their physiological function, their role in pathology with a special focus on the amyloid formation process, and possible mechanisms of intervention.

## 2. Transthyretin

**Figure 2:**
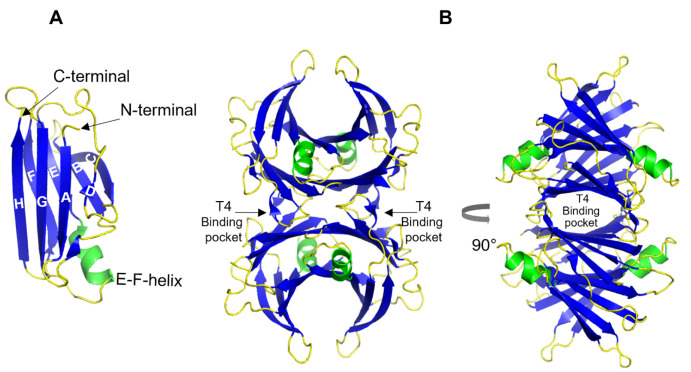
3D ribbon diagram of the native human TTR protein. (**A**). TTR monomer with eight β-strands A-H, and an α-helix between E and F strands. (**B**). TTR tetramer in two different orientations and indicated thyroxine-binding pocked. Key features are colored as β-strands in blue and α-helices in green. The figures are created from PDB-ID: 1DVQ.

TTR is a 55 kDa homotetrameric protein circulating in the plasma and CSF. Its main physiological function is to transport thyroid hormone thyroxine (T4) and retinol-binding protein. TTR is produced mainly in the liver [49] wherefrom it is secreted into the blood with concentration levels ranging between 4–7 µM. In the CSF, the concentration of TTR is about 20 times lower and it is primarily synthesized in the choroid plexus [50]. TTR is also expressed by the islet of Langerhans of the pancreas [51] as well as within the retina of the eye [52]. TTR gene expression can also be found in Schwann cells, dorsal root ganglia [53], and cortical and hippocampal neurons in response to Aβ-induced stress [54]. The structure of TTR is well studied and was first solved by X-ray crystallography in 1978 [55] (Figure 2). Each TTR monomer consists of 127 amino acid residues forming eight β-strands named A-H and one short α-helix between E and F strands [55,56] (Figure 2A). The β-strands CBEF and DAGH are arranged into two β-sheets. Two monomers form a dimer via hydrogen bonds between antiparallel H-H’ and F-F’ strands. The functional tetramer is formed from two dimers predominantly by hydrophobic interactions between the residues of A-B and G-H loops, producing a central hydrophobic pocket with two binding sites for thyroxin hormone [55,56] (Figure 2B). Both monomer–monomer and dimer–dimer interactions are important for the stability of TTR [57].

TTR also possesses intrinsic amyloidogenic features and its aggregation is linked to several amyloid disorders including senile systemic amyloidosis (SSA), familial amyloid cardiomyopathy (FAC), and familial amyloid polyneuropathy (FAP)—a hereditary disease variant caused by one of over 100 known point mutations in the *ttr* gene [58,59,60]. The initial and rate-limiting step in TTR aggregation is the dissociation of the native tetramer into monomers that subsequently undergo conformational changes forming aggregation-prone intermediates [61,62,63,64,65].

Paradoxically, being an amyloidogenic protein itself, it was found that TTR has an inhibitory effect on AD-associated Aβ aggregation via direct interaction with the latter [66,67,68,69,70,71]. This discovery suggested a protective role of TTR against AD, where a possible failure of these properties could serve as a precondition for the disease development [32] (the role of TTR in AD was recently reviewed by Giao et al. [72]). This hypothesis is further supported by clinical data showing a significantly reduced level of TTR in the CSF of AD patients compared to healthy individuals [73,74]. Moreover, the severity of the disease was inversely correlated with the concentration of TTR [74]; however, no correlation was found between the variants of TTR and AD [75].

Since then, numerous studies have confirmed the protective effect of TTR in animal models of AD. Particularly, overexpression of human TTR in the widely used AD transgenic mouse model APP23 prevents cognitive impairment [76]. Stein et. al. showed that transgenic Tg2576 mice that overexpress mutant amyloid precursor protein (APP) exhibit upregulation of TTR expression, and chronic infusion of an anti-TTR antibody into the hippocampus of these mice exacerbates Aβ pathology, as the concentration of free TTR is decreased [77]. In addition, hemizygous TTR deletion in APPswe/PS1deltaE9 mice resulted in earlier deposition of Aβ fibrils compared to control mice [78]. It has been shown that the expression level of TTR, similar to Aβ-degrading neprilysin, is controlled by the intracellular domain of APP [79]; thus, overexpression of APP in Tg2576 mice could mediate the up-regulation of TTR [77].

The protective effect of TTR against Aβ-induced cytotoxicity has also been investigated in cellular models [35,67,80,81,82]. Together, these findings suggest a functional connection between Aβ and TTR, and their interactions have been studied extensively in vitro [33,34,37,69,70,83]. The results support an inhibiting effect of TTR on Aβ amyloid formation. However, in this context, there are several controversies in the field. By using nanoparticle tracking, Yang et al., showed that partially aggregated Aβ binds to TTR more efficiently than the monomeric or heavily aggregated form of Aβ, and monomeric TTR binds more Aβ than tetrameric form [84]. The authors suggest a mechanism where the EF-strand helix/loop of TTR tetramer acts as a sensor in the presence of Aβ oligomers, the binding of which triggers destabilization of TTR tetramer and exposure of inner hydrophobic sheets, thus increasing the oligomer binding capacity and arresting their further aggregation [84]. In contrast to this, Li et al., demonstrated that Aβ monomers bind to the ligand-binding pocket of TTR, thus sequestering monomers from the oligomer formation process [34]. At the same time, they also suggest that both tetrameric and monomeric TTR can bind Aβ oligomers, with greater efficiency for TTR monomers in vitro [34]. In support of this, some data suggest an inverse correlation between TTR stability and the inhibitory potential on Aβ aggregation in vitro [69], and that TTR dissociation into monomers is required for the inhibition of Aβ-induced cytotoxicity, while stable tetrameric variants are nonefficient [82]. In contrast, others show that unstable TTR binds poorly to Aβ [85] and that the stability of tetrameric TTR is critical in Aβ clearance by assisting its internalization and degradation in lysosomes [86]. Moreover, oral administration of the TTR stabilizing drug iododiflunisal promotes Aβ clearance, leading to reduced Aβ deposition and improved cognitive functions in AD mouse models [87,88]. In this context, it is interesting to note the work by Li et al. [34], where they show that in vitro the kinetically rather unstable TTR-V122I variant is more efficient in inhibiting Aβ aggregation compared to the more stable TTR-T119M and TTRV30M. However, even the most kinetically stable variant can suppress Aβ aggregation at a TTR:Aβ ratio corresponding to 1:5. The authors also suggest that the observed in vitro interactions of TTR monomers with Aβ oligomers might not reflect in vivo situations where the tetrameric form of wild-type TTR is the dominating variant and dissociation of TTR tetramer is not necessary for its inhibitory effect [34]. Given that in vivo the extracellular Aβ concentration is much lower (within high picomolar-low nanomolar range) and TTR concentration is ranging from 0.25–0.5 μM in CSF and 3–5 μM in human serum, this should provide sufficient condition for performing its protective activity against Aβ aggregation. However, studies on AD patients revealed an inability of TTR to bind its natural ligand [89] and a decreased ratio of folded/monomeric TTR in plasma [86], which suggests a deficit in functional TTR molecules. Thus, stabilizing the TTR could represent one of the possibilities in AD therapy which is discussed in a recent review by Saponaro et al. [90].

**Figure 3:**
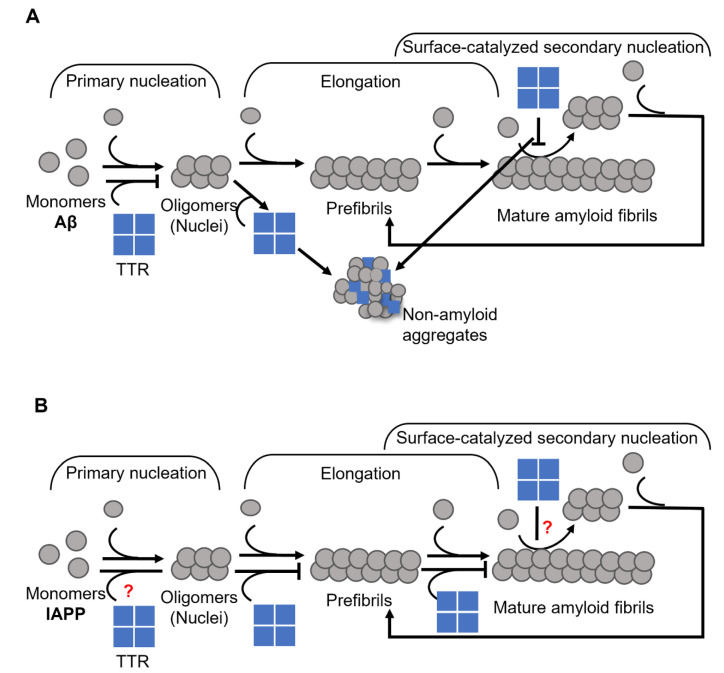
Schematic presentation of suggested mechanisms for TTR interference with (**A**) Aβ, where TTR targets both primary and secondary nucleation and re-directs the reaction towards the formation of non-amyloid aggregates (modified from Nilsson et al., 2018 [37]), and (**B**) IAPP, where TTR specifically targets the elongation process (modified from Wasana Jayaweera et al., 2021 [91]) amyloid formation.

In a previous study, we showed that the inhibition of Aβ fibril formation by TTR primarily involves the nucleation stage and results in the formation of ThT- negative non-amyloid aggregates [37]. However, we also noted that TTR does not affect the process of elongation (Figure 3A). More recently Ghadami et al., reported that TTR can inhibit both primary and secondary nucleation of Aβ aggregation, at the same time confirming our observation on the inability of TTR to affect the elongation [70]. Interestingly, by modulating the conditions of nucleation as well as the concentration of TTR, essentially a complete conversion into non-amyloid amorphous Aβ assemblies could be acquired [37].

Besides the inhibitory effect on Aβ aggregation, there is also evidence that TTR may interfere with other amyloid-forming polypeptides. Recently, Pate et al., showed that both engineered monomeric TTR and cG8 cyclic peptide, which is based on an Aβ-binding domain of TTR, efficiently inhibits amylin aggregation, although both did not affect α-synuclein [92]. Amylin or islet amyloid polypeptide (IAPP) aggregation is associated with type 2 diabetes, where IAPP amyloid affects the islets of Langerhans accompanied by a decreased number of β-cell [93,94,95]. Normally TTR is expressed by pancreatic α-cells [96], and in much lower levels by β-cells [51]. Interestingly, increased TTR levels were found in β-cell in type-2 diabetes patients’ pancreatic islets with heavy amyloid deposits [51].

In a recently published work, we showed that TTR affects the process of IAPP amyloid formation by targeting elongation [91] (Figure 3B), which is in stark contrast to its effect on Aβ (see Figure 3A,B) [37,70]. We also showed that the efficacy of TTR displays an inverse correlation with thermodynamic stability, while no such correlation could be noted regarding the rate of dissociation of the tetramer. However, the addition of TTR-stabilizing drugs partly suppresses its efficiency [91] in contrast to the suggested beneficial effect on Aβ [87,88], hence supporting that a partial unfolding is required for its inhibitory actions.

These findings suggest that TTR can have an important contribution in regulating the aggregation of certain amyloidogenic polypeptides in vivo. The interaction of TTR with other amyloidogenic proteins, such as bacterial CsgA [97] and HypF-N [35], also indicates its generic nature as an anti-amyloidogenic molecular chaperon.

However, the experimental data available for Aβ and IAPP suggest that although TTR has a common anti-amyloidogenic property on these peptides, the underlying mechanisms of inhibition might be different, and further investigations are needed to gain a better understanding of these mechanisms.

The potential use of TTR as a target to improve its anti-amyloidogenic properties should, however, be handled with care due to its intrinsic amyloidogenic features. Clinical means to improve its anti-amyloidogenic features should consequently also make sure to not enhance its intrinsic amyloidogenic features. The role of TTR as an amyloid-interfering protein may however already be put to the test. The recent development within the techniques of RNA interference has resulted in clinically approved treatments for FAP where the endogenous TTR produced in the liver is suppressed by more than 90% [98]. Here the reverse question is raised where lack of TTR instead potentially may enhance the formation of other amyloids such as Aβ or IAPP.

## 3. Apolipoprotein E

Apolipoprotein E (ApoE) is a 34 kDa lipid-binding and transport protein found in both serum and CSF. There are two main sources of production of ApoE. In the periphery, the majority of circulating ApoE is synthesized by the liver [99], while in the central nervous system (CNS), it is mainly produced by glial cells [100]. Peripheral ApoE plays a critical role in lipid metabolism by transporting lipoproteins into the lymph system and blood and maintaining the cellular uptake of lipoproteins via its primary receptor LDLR (low-density lipoprotein receptor) [101,102]. ApoE in the CNS promotes the transport of lipids into neurons, thus participating in neuronal maintenance, synaptic remodeling, repair, and lipid homeostasis in CNS [103,104]. Human ApoE exists in three isoforms—ApoE2, ApoE3, and ApoE4, which are encoded by the three alleles ε2, ε3, and ε4, respectively [105]. The dominating isoform in the global population is ApoE3 with an allele frequency of 65–70%, while the ApoE4 variant is found within 15–20% of the population [106]. The discovery of a strong genetic linkage to AD, where the ApoE4 variant was found to be a high genetic risk factor for the development of the disease [39,105,107], brought this protein into focus. Heterozygous carriers of ε4 allele have about a 2 to 3-fold higher risk of developing AD compared to homozygous ApoE3 carriers, while for homozygous ε4 allele carriers the risk increases about 10-fold compared to non- ε4 carriers [107] and 40–65% of all AD patients are carriers of at least one copy of ApoE4 [108]. In contrast, ApoE2 is considered protective [105] and the prevalence of AD development within this group is lower as compared to ApoE3.

**Figure 4:**
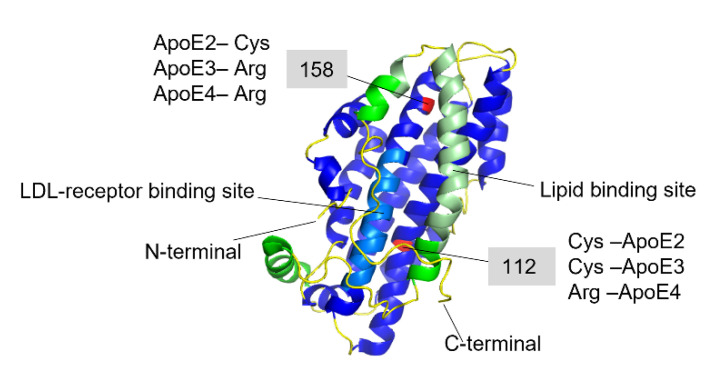
Full-length structure of Apolipoprotein E3 (PDB-ID: 2L7B). The N-terminal domain is colored in blue where the light-blue region is the LDL-receptor binding site, and the C-terminal domain is colored in green, where the light green region is the lipid-binding site. The red-colored residues at 112 and 158 positions are the varying residues between the three ApoE variants.

ApoE has been found co-localized with Aβ-amyloid plaques with relative prevalence of ApoE4 > ApoE3 > ApoE2 [109,110]. These results suggested that ApoE might play a specific role in Aβ amyloid formation and related AD pathology, which has been explored in numerous studies [39,40,41,42,110,111,112,113,114,115,116,117]. The amino acid differences between the three isoforms are only at positions 112 and 158, where ApoE2 has cysteine residues at both positions, ApoE3 has a cysteine at positions 112 and an arginine at position 158, and ApoE4 has arginine residues at both positions (Figure 4) [105,118,119].

These seemingly small differences in the sequence affect the ApoE receptor and lipid binding abilities and functionality [105,120,121,122]. The ApoE molecule consists of two distinct structural N- and C-terminal domains linked together by an unstructured hinge region, which is supporting the mobility of the structure. The N-terminal domain represents a bundle of four α-helixes, comprising positions 1–191, where the LDL-receptor binding region is located (residues 134–150) [105,123]. The C-terminal amphipathic α-helical domain consists of residues 210–299 with a largely exposed hydrophobic surface, where the lipid-binding region is located within the residues 244–272 [105,123]. When ApoE binds to the lipids the whole molecule undergoes conformational changes to adopt a biologically active form, which is necessary for recognition and binding to the LDL-receptors and internalization [124]. Although the isoform-specific residues are located within the N-terminal domain, the preferences for lipid and lipoprotein binding are different between the three ApoE isoforms. ApoE2 and ApoE3 variants are mostly associated with high-density lipoproteins (HDL), while ApoE4 prefers low-density lipoproteins and very-low-density lipoproteins (LDL, VLDL) [125]. These preferences are most likely attributed to differential conformational changes of the whole ApoE molecule provided by the residues at positions 112 and 158. The X-ray crystallography analysis showed that in the dominating isoform-ApoE3 (associated with normal lipid and lipoprotein metabolism), there is a salt bridge formation between the Arg-158 and Asp–154. This salt bridge seems to be critical for LDL-receptor recognizing and binding [106,126]. In the ApoE2 variant, Arg to Cys substitution at position 158 leads to disruption of the natural salt bridge and formation of a new bridge between the Asp154-Arg150. This in turn impairs the binding ability of ApoE2 to the LDL-receptor, which is associated with type III hyperlipoproteinemia [126]. In ApoE4, Arg112 causes a rearrangement of the N-terminal Arg61 side chain, which is exposed towards Glu255 in the C-terminal domain leading to a salt bridge formation between these two residues [127]. This interaction between the two domains is responsible for the preferential binding of ApoE4 to VLDL [122,127]. In general, ApoE4 is shown to have higher affinity to the lipids and lipoprotein particles, regardless of their size [122]. On the other hand, it has been suggested that the unique interaction between the two domains leads to accelerated catabolism of ApoE4 and consequently, elevated cholesterol and LDL levels in plasma [122,127].

Lipid binding to the C-terminal is a requirement for the ApoE molecule to gain LDL-receptor binding ability. When bound to the lipids, the C-terminal α-helices are oriented perpendicularly to the acyl chain of the lipids [128,129]. For the N-terminal domain, several models were suggested to adapt for lipid-bound ApoE. Calorimetric studies showed that ApoE gains an extended conformation due to the four-helix bundle opening, which allows wrapping around the lipid bilayer [129]. In contrast to this, other studies suggested a hairpin conformation of ApoE in the lipid-bound state [130,131,132]. In a more recent study by Henry et al. [124], it was proposed that both the open and compact hairpin conformations may co-exist in a dynamic equilibrium, which is shifted to the opened hairpin model in the presence of the LDL-receptor. Thus, both conformations may be part of a regulation mechanism of ApoE function at the surface of lipids [124].

Further, differences in stability between the ApoE variants indicate that ApoE4 is less stable and that it also may aggregate into amyloid-like fibrils [133,134]. In contrast, a recent study revealed no significant differences between the recombinant ApoE variants at the structural or conformational level [120].

It has been shown that ApoE can directly interfere with the Aβ aggregation process by inhibiting or slowing down fibril formation [39,40,41,42,113,116,135]. However, the impact of this interaction on disease progression remains unclear. Several studies suggest that ApoE acts as a pathological chaperone, although to different extents depending on the variant. For example, studies on AD mouse models revealed that all ApoE-carrying AD mice developed amyloid plaques with the highest load observed in ApoE4 animals, while the complete knockout of the ApoE gene significantly reduced plaque formation and other signs of disease [109,136]. In agreement with this, administration of ApoE-specific antibodies promoted efficient clearance of Aβ plaques [137]. In contrast, several studies indicate that ApoE plays an important role in the degradation and clearance of Aβ amyloid [110,111,138,139]. For example, it has been shown that ApoE protects human pericytes against Aβ-induced cytotoxicity [112] and maintains a receptor-mediated in-pericyte clearance of Aβ aggregates [115], with significantly weaker effects detected for ApoE4 compared to the other variants [112,115]. Interestingly, cells with the ε4 genotype expressed lower levels of ApoE and exhibited a higher vulnerability to Aβ toxicity compared to cells with ε2 or ε3 genotype [112,140]. Moreover, a low level of ApoE has been considered as a general risk factor for AD irrespective of isoform [141,142]. Therefore, low ApoE expression encoded by the ε4 allele could explain its association to AD pathology as in this case the levels of functional ApoE4 would be insufficient to perform its protective function. However, reports on the levels of ApoE in AD also remain controversial. Several studies link low ApoE levels in plasma to an increased risk of AD and dementia [143,144], while others argue that the low levels are specifically associated with the ApoE4 isoform and that there is no difference in ApoE levels in AD vs non-AD individuals [145]. Similarly, conflicting results are reported for CNS ApoE levels in AD, shown to be either decreased [146,147,148], increased [149,150,151], or unchanged [145,152].

In support of a protective role of ApoE, a recent case report described an individual carrying the ApoE3 Christchurch variant with no signs of AD, despite expectations to develop early-onset AD due to an aggressive familial presenilin mutation [153]. These data further highlight the clinical importance of ApoE both in modulating the amyloid formation and disease progression, as well as implying that ApoE generally has a protective role against amyloid formation and toxicity, with a loss of function for ApoE4.

**Figure 5:**
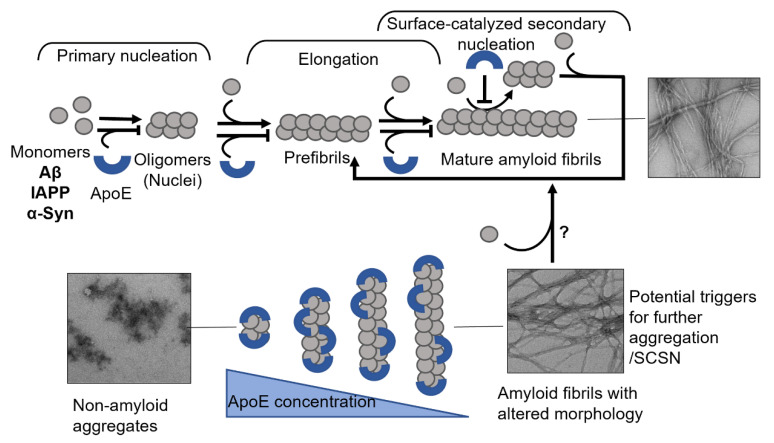
Mechanisms of ApoE interference with amyloid fibril formation. The schematic presentation in the upper panel shows that ApoE can target multiple steps of amyloid formation, including primary nucleation, elongation, and secondary nucleation. The lower panel shows the effect of ApoE concentration in interfering with amyloid formation process, where the higher concentration can convert the amyloid protein into non-amyloid aggregates and the lower concentrations lead to the production of amyloid fibrils with altered morphology.

In vitro, the direct interaction of ApoE with Aβ has an inhibitory effect on the amyloid formation process [41,42,83,135]. Our group recently reported that ApoE targets both the process of Aβ nucleation and fibril elongation, and effectively prevents the maturation of amyloids in a concentration-dependent manner [135] (Figure 5). This effect is pronounced even at highly substoichiometric ratios of ApoE:Aβ with low nM concentrations of ApoE and about 1000 times excess of Aβ. This finding indicates that ApoE most likely binds to Aβ assemblies rather than monomers (Figure 5).

Besides Aβ, only a few studies examined interactions of ApoE with other amyloid-forming proteins [154,155,156]. Lei et al., showed earlier that ApoE can directly interact with IAPP in vitro, and the effect on amyloid formation is dependent on ApoE concentration, with higher concentrations promoting IAPP aggregation and lower concentrations inhibiting it [154]. In contrast to this, our recent studies indicate that ApoE efficiently suppresses IAPP amyloid formation in a concentration-dependent manner starting already at highly sub-stochiometric concentrations. Upon increasing ApoE concentration in the reaction, we observed the formation of mainly ThT-negative assemblies with amorphous morphology [156]. Interestingly, in this study, we saw no difference in amyloid interfering ability between the three ApoE isoforms. However, we showed that ApoE protects the cells from IAPP-induced toxicity with the weakest effect observed in the ApoE4 variant. The difference between the protective effects of ApoE isotypes is especially pronounced at low nM concentrations and longer treatment time, where ApoE2 and ApoE3 still efficiently protect the cells, but where ApoE4 almost completely loses this ability [156].

Another possible target of ApoE is α-synuclein, a protein involved in dementia with Lewy bodies, including PD. However, the existing data are scarce and controversial. Gallardo et al., showed that overexpression of human α-synuclein in transgenic mice induces neurodegeneration and leads to a massive increase of ApoE and Aβ [157]. While this could be a defensive response directed to reduce α-syn aggregation and protect from PD, the authors showed that the elevation of ApoE levels is accompanied by Aβ aggregation [157]. Another study showed that at very low concentrations ApoE can promote α-synuclein aggregation, while higher concentrations have an inhibitory effect on the aggregation process [155].

These and our data [135,156] indicate the importance of the concentration factor of an amyloid inhibiting protein, where simply insufficient inhibition could lead to the formation of more pathological species (see Figure 5, where in the presence of lower concentrations of ApoE, the generated fibrils adopt a more diffuse morphology) and trigger further aggregation of an amyloidogenic protein. In response to early amyloid formation, cells could possibly increase ApoE expression as a protective measure to halt the aggregation process. Failure in raising ApoE levels could then lead to a sustained amyloid formation process. Carrying the ApoE4 variant additionally increases this risk due to its intrinsic properties and weaker protective ability, which can be observed on cellular models [112,156].

Several ApoE-based therapeutic approaches have been suggested particularly for AD and summarized in a recent review [158]. Among these approaches are gene editing and gene therapy directed to recalibrate ApoE function via CRISPR-mediated or adeno-associated virus-mediated gene delivery. By those methods, it is possible to edit *ApoE4* allele to *ApoE3*, replace *ApoE4* by protective *ApoE2* or overexpress *ApoE2* in *ApoE4* carriers, which could play a compensatory role for loss of function *ApoE4*. Other suggested approaches include restoring or tuning the function of ApoE via enhanced lipidation or targeted modification of *ApoE4* structure. Alteration of ApoE levels is also considered as a potential disease-modifying therapy [158].

## 4. Clusterin

Clusterin, also known as Apolipoprotein J, is a well-conserved, 50 kDa heterodimer protein [159]. It is mainly synthesized in the liver, prostate, and ovaries from where it is secreted into the plasma [160,161]. The concentration of circulating clusterin in human plasma is ~100 μg/mL, while it is present at only ~2 μg/mL in the CSF [162,163]. Clusterin is primarily expressed and released from astrocytes in the CNS [159].

**Figure 6:**
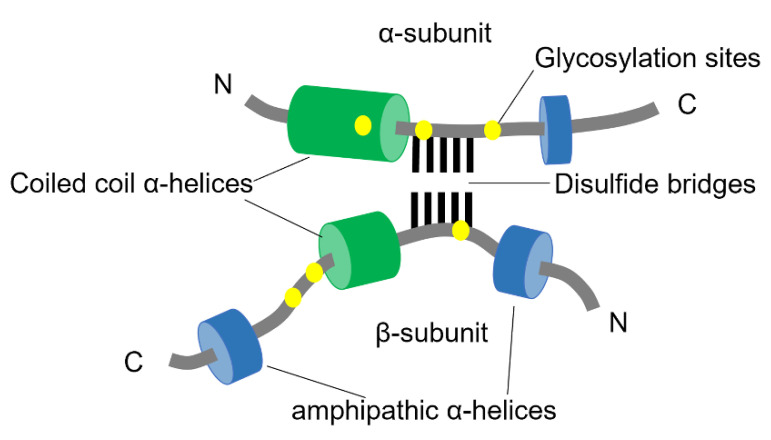
Schematic structure of Clusterin. The two subunits of clusterin are assembled anti-parallel resulting in a heterodimeric molecule. The cysteine-rich centers are linked by five disulfide bridges (black lines) and are surrounded by two predicted coiled-coil α-helices (green) and three predicted amphipathic α-helices (blue). The N-linked glycosylation sites are indicated as yellow spots (adapted from Jones et al., 2002 [166]).

The structural properties of clusterin are not well understood, probably due to its aggregation-prone nature. It is synthesized as a polypeptide comprised of 449 amino acids with a 22-residue signal peptide at the N-terminus. The protein is cleaved between amino residues 205 and 206 to generate two different subunits that remain connected via disulfide bonds [164]. Furthermore, intrinsically disordered domains have been predicted at the N- and C- termini of both subunits [164,165] (Figure 6).

The mature clusterin is highly N-glycosylated and due to a variable degree of glycosylation, it displays a mass between 58.5–63.5 kDa when analyzed by mass spectrometry.

Together with ApoE, clusterin is one of the most abundant apolipoproteins in the brain [159,166]. It participates in multiple biological processes in the body, including removal of cellular debris [167], regulation of programmed cell death [168], lipid transport [169], and exhibits a chaperon-like activity in several protein folding processes [169,170,171]. As mentioned, clusterin is mainly a secreted protein, but there are cytoplasmic and nuclear forms that are activated under several stress conditions [172].

Clusterin was associated with AD after identifying multiple genetic variations (single nucleotide polymorphisms) in its *CLU* gene in late-onset AD patients in independent studies [173,174,175]. However, the role of clusterin in AD remains unclear. In contrast to TTR and ApoE, the levels of clusterin are consistently increased in blood plasma [176], CSF [177], and in the affected brain areas [178,179] of AD patients. Interestingly, the increase in clusterin levels was found to be proportional to the number of apoE4 alleles in the brain of AD subjects, where low levels of ApoE could be compensated by elevated levels of clusterin [146].

The involvement of clusterin in different signaling pathways, relevant for AD, is discussed in a recent review [159] where the dual role of clusterin is elucidated. On one hand, clusterin has a protective role in AD by improving autophagy, regulating the proliferation of neuronal precursors, and inhibiting TNFα-induced apoptosis in Akt signaling pathway. On the other hand, clusterin can activate Wnt-PCP signaling pathway and cause JNK activation, which mediates tau phosphorylation and Aβ neurotoxicity.

Clusterin is found colocalized with Aβ plaques and considered as one of the key regulators of Aβ metabolism. However, the mechanisms of interaction between Aβ and clusterin are not fully understood and studies often report controversial results. For example, clusterin was reported to have a protective role by maintaining Aβ clearance [180], reducing its toxicity by inhibiting amyloid assembly [43], and preventing senile plaque formation [181]. In contrast to this, clusterin has been reported to promote fibrillogenesis [182] and the knock-out of the *ApoJ* gene reduced fibrillary Aβ, thus preventing Aβ-induced neuronal cell death [159]. However, it is noteworthy that although clusterin-knockout AD mice have less Aβ accumulation and plaque formation in the cerebral cortex and hippocampus, they develop cerebral amyloid angiopathy (CAA) as Aβ accumulates in the cerebrovasculature [183]. In a healthy brain, most of Aβ is cleared by vascular transport across the blood–brain barrier (BBB), and only a small fraction of Aβ is removed via perivascular clearance mechanism [184]. The clearance of Aβ across the BBB is mediated by clusterin by binding and subsequent transport via low-density lipoprotein-related protein 2 (LRP2) [44]. In the absence of clusterin, a proper clearance of Aβ fails and is instead shifted to the perivascular pathway [183].

**Figure 7:**
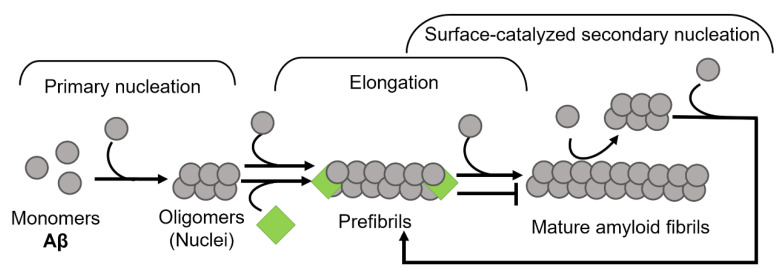
Schematic illustration of clusterin on the aggregation kinetics of Aβ. Clusterin perturbs the amyloid formation process by binding to fibril ends, thus inhibiting the elongation phase (modified from Scheidt et al., 2019 [186]).

The amyloid-interfering property of clusterin depends on the ratio of clusterin to substrate in vitro. At high clusterin levels and a molar ratio of 1:10 of clusterin to substrate, clusterin exhibits strong anti-amyloidogenic properties by inhibiting fibril formation, as well as protecting the cells against amyloid-induced cytotoxicity. However, at much lower concentrations of clusterin and with an excess of the amyloidogenic protein, clusterin promotes the amyloid formation, and thus enhances cytotoxic effects caused by these clusterin-amyloid complexes [185]. The affinity of clusterin to the substrate is rather conformation-specific than substrate-specific as it binds to intermediate species during the fibril-forming process (Figure 7) and can interfere with a wide range of amyloid-forming proteins including Aβ, α-syn, calcitonin, and the short coiled-coil β [43,185].

The inhibitory effect of clusterin has been shown also on tau fibrillization in vitro, and the protective effect of clusterin against tau pathology in an AD mouse model [187]. This supports the hypothesis that clusterin can have a generic protective role against protein aggregation. However, it is not clear whether the modulatory effects on Aβ aggregation and tau pathology by clusterin occur independently during AD or if it is linked with a common mechanism.

From the experimental data available today one can conclude that the important factor in regulating amyloid formation may not only be the absolute levels of clusterin but rather the ratio of clusterin/amyloid-forming protein. Even the significantly increased protein production, which is most likely a protective response of the body to the elevated harmful amyloidogenic protein, can be consumed rapidly and competed by progressively elevating levels of amyloidogenic protein. As it has been shown on APP23 mice, peripheral administration of clusterin reduces insoluble Aβ and CAA load in the brain [188] and thus could have been considered as a therapeutic intervention in the human clinic.

Taken together, clusterin has a good therapeutic potential for AD and possibly, other amyloid-related disorders. Detailed understanding of the role of clusterin in different cell signaling pathways associated with these pathologies and the mechanisms of interaction with the amyloid-forming proteins will help develop approaches that can enhance beneficial properties of clusterin which will lead to reversion or normalization of pathological processes.

## 5. Brichos

**Figure 8:**
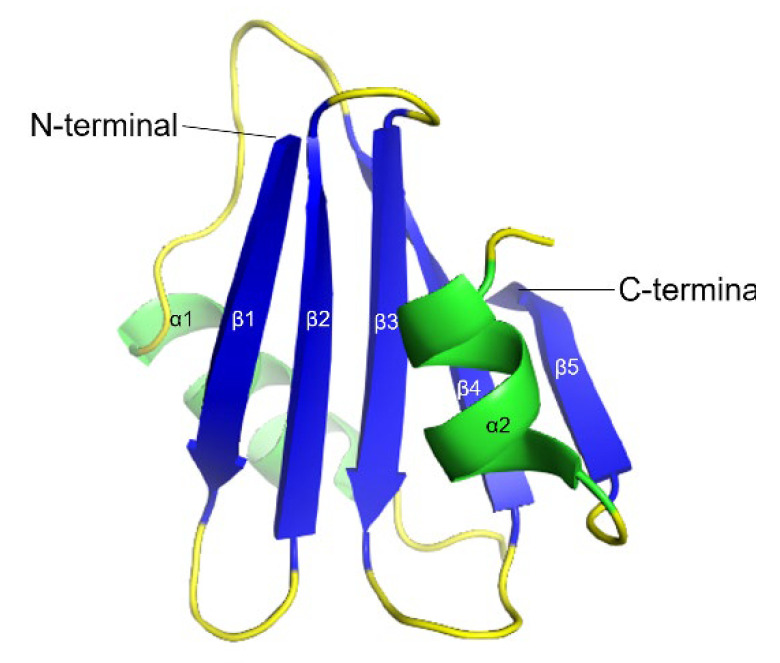
3D ribbon backbone conformation of proSP-C BRICHOS domain (PDB-ID: 2YAD). The five β-strands (β1–β5) are shown in blue and the two α-helices (α1 and α2) in green.

BRICHOS is a 100 amino acid residue protein domain found in 12 protein families including over 300 proteins (Figure 8) [189]. The name BRICHOS was derived from its initial discovery on the proteins called Bri2, Chondromodulin-I, and proSurfactant protein C (proSP-C). Bri2 is expressed in neural tissue and related to familial British dementia (FBD) and familial Danish dementia (FDD). Chondromodulin-I is associated with chondrosarcoma, while ProSP-C and its mature form SP-C are linked to interstitial lung disease [190,191]. BRICHOS family proteins have an overall conserved architecture containing five regions: an N-terminal cytosolic part, a hydrophobic transmembrane part, a linker region, BRICHOS, and C-terminal, with the exception for proSP-C which is lacking the C-terminal [45]. Although the residual conservation is low among the regions from the different protein families, which indicates the substantial differences in functionalities of these proteins, the BRICHOS domain is the most conserved within all families (51–83% average pairwise percent identities), suggesting a common function of this region for all families [189]. Moreover, all BRICHOS domains have three strictly conserved residues, one aspartic acid and two cysteines. These cysteines form a disulfide bridge in proSP-C BRICHOS and are predicted to be the same for BRICHOS from other families [66].

Proteolytic processing of BRICHOS proteins results in the release of different peptides from C-terminal, such as Bri23 functional peptide from Bri2. Further processing can also release the BRICHOS domain to extracellular space [191,192].

Among the BRICHOS containing proteins, proSP and BRI families are the most studied for BRICHOS function. It is believed that the physiological role of BRICHOS is acting as a chaperone during the protein folding process, preventing them from misfolding and aggregation. ProSP-C and Bri2 contain an amyloidogenic segment and it is hypothesized that BRICHOS domain prevents these segments from forming aggregates [193,194]; however, the anti-amyloidogenic properties of BRICHOS are not limited to its precursor proteins and have been extensively studied in relation to AD-associated Aβ aggregation [45,46,48,66,193,195].

ProSP-C, a 21kDa transmembrane protein, is expressed and secreted from the pulmonary alveolar type II cells and eventually cleaved to form a 35 residue of lipophilic mature peptide SP-C in the alveolar space to stabilize surfactants at low lung volumes [196,197,198]. Under physiological conditions, the poly-Val segment of the native proSP-C is prone to form amyloid aggregates. The BRICHOS prevents the poly-valine folding to β-sheets and forms a stable α-helix structure [194,199]. Mutations of the proSP-C BRICHOS domain occurring either within the linker region or in the BRICHOS sequence leads to amyloid aggregations of SP-C that causes interstitial lung disease and lung fibrosis [191,198,200] The most common mutation is I73T in the linker between the transmembrane and BRICHOS regions. SP-C isolated from lung surfactant forms amyloid aggregates in vitro; however, when co-incubated with proSP-C BRICHOS these aggregates are not formed [201].

Among the BRICHOS proteins, the Bri2 and Bri3 of the BRI family play a significant role in the prevention of neurodegenerative diseases [47,193,202,203]. These proteins are highly expressed in the brain. The Bri2 BRICHOS domain is shown to bind to Bri23 peptide that is released from the C-terminal, preventing its aggregation [47,193]. Mutations in Bri2 result in extended C-terminal, the cleavage of which gives rise to two different 34 residue peptides ABri or ADan. These peptides form amyloid-like deposits in the brain and are associated with FBD and FDD, respectively [204]. They also cause cell toxicity and affect synaptic plasticity of neurons similar to AD-related Aβ [205]. However, there are no data available about an interaction between the BRICHOS domain and ABri or ADan peptides. In addition, at least in the case of FDD, an increased APP processing and significantly increased Aβ suggest that AD and FDD can share a common mechanism [206]. It is noteworthy that at physiological conditions, both Bri2 and Bri3 interact with APP preventing its pathological cleavage and halting the release and aggregation of Aβ peptide [207,208]. It is still not clear whether ABri or ADan alone can cause the disease or a loss of function of the Bri2 protein, and thus affected processing of APP and Aβ aggregation also could contribute to the pathology [204,209,210,211].

An increased overall level and deposition of Bri2 in amyloid plaques of AD brains has been observed at early stages of AD development, which is accompanied by the decreased presence of Bri2-APP complexes, suggesting a loss of function of Bri2 during AD [48]. In contrast to Bri2, the overall levels of Bri3 are shown to be decreased in AD, although the deposition with the amyloid plaques is similar for both Bri2 and Bri3 [202]. BRICHOS from both Bri2 and Bri3 interact with Aβ in neurons and inhibit Aβ fibrillization in vitro, but Bri3 shows less efficiency compared to Bri2 [202,203], suggesting a different role for Bri2 and Bri3 BRICHOS in Aβ pathology.

Studies on transgenic Drosophila showed that co-expression of Aβ42 and BRICHOS domain in the CNS delays the aggregation of Aβ42 and significantly improves both lifespan and locomotor function compared with only Aβ42 expressing flies [212]. Moreover, BRICHOS increases the ratio of soluble:insoluble Aβ42, binds to Aβ aggregates, and efficiently reduces the neurotoxic effects of Aβ42 in the fly brains. [195,212]. However, the BRICHOS from Bri2 exhibits more efficiency than the BRICHOS from proSP-C [195].

In vitro, recombinant proSP-C BRICHOS and Bri2 BRICHOS directly interact with Aβ42, significantly reducing its aggregation already at substoichiometric levels of BRICHOS [213].

Interestingly, different quaternary structures of BRICHOS affect qualitatively different aspects of protein misfolding and toxicity [214]. Particularly, Bri2 BRICHOS monomers potently prevent Aβ-induced neuronal network toxicity, while dimers strongly suppress Aβ fibril formation, and higher molecular weight-oligomers efficiently inhibit non-fibrillar protein aggregation [214]. It has been shown also that Bri3 BRICHOS forms more and larger oligomers and prevents non-fibrillar protein aggregation better than Bri2 BRICHOS oligomers, which explains its lower efficiency in suppressing Aβ42 fibrilization compared to Bri2 BRICHOS [203].

**Figure 9:**
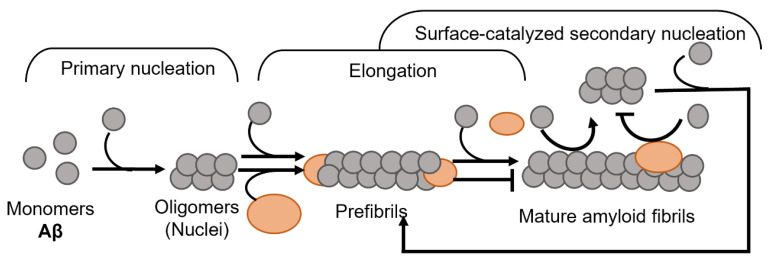
Mechanism of BRICHOS domain in amyloid fibril formation. BRICHOS domain binds both to the ends as well as laterally on fibrils and thereby inhibits both elongation and secondary nucleation events (modified from Arosio et al., 2016 [215]).

The mechanism by which BRICHOS inhibits Aβ amyloid formation is explored in several studies. Cohen et al., showed that BRICHOS specifically inhibits surface catalyzed secondary nucleation by binding to the surface of Aβ42 fibrils [213]. This interaction breaks the catalytic cycle and thus suppresses the continuous production of neurotoxic oligomers. The aggregation reaction instead is redirected towards end-point mature fibril formation, which involves only primary nucleation and elongation [213]. Later it has been shown that while the BRICHOS from proSP-C inhibits only secondary nucleation, the BRICHOS from Bri2 targets both secondary nucleation and fibril elongation of Aβ42 by binding to both fibril surface and fibril ends, respectively [215] (Figure 9). This difference between the two BRICHOS is reflected in the study on Drosophila, where the protective potential of Bri2 BRICHOS against Aβ-induced pathology was higher compared to proSP-C BRICHOS [195]. These findings suggest that although BRICHOS from different precursor proteins share common features, they show, however, selectivity towards their amyloidogenic substrates with an optimal efficiency corresponding to the areas of physiological activity.

Recent studies on mice showed that peripherally administered recombinant BRICHOS can pass over the BBB [216], and the delivery can be enhanced by focused ultrasound and microbubbles [217]. The delivered BRICHOS is uptaken by hippocampal neurons. Moreover, a specifically designed Bri2 BRICHOS mutant (R221E) that forms stable monomers selectively blocks the production of toxic oligomers during Aβ42 aggregation. In the presence of this BRICHOS variant, the oligomers of wild type Bri2 BRICHOS are partly disassembled into monomers, leading to potentiated prevention against Aβ42-induced toxicity [218]. These results could have therapeutic significance in suggesting that the delivery and the activity of endogenous molecular chaperones can be modulated to enhance their anti-amyloid properties.

The inhibitory activity of BRICHOS has recently been explored also on IAPP aggregation and toxicity [219]. The study shows a high expression of Bri2 in human pancreatic islets and β-cells, as well as co-localization of Bri2 with IAPP both intracellularly and in the islet amyloid deposits from type 2 diabetes patients. Moreover, BRICHOS showed a strong inhibitory effect on IAPP amyloid formation in vitro by targeting secondary nucleation and redirecting the reaction towards formation of amorphous aggregates. It also reduced IAPP-induced toxicity both in cell lines and in a Drosophila model [219]. These findings extend the knowledge on BRICHOS role and properties and suggest that it can be an important therapeutic target not only for AD or type 2 diabetes but probably for other amyloidoses, which need further investigations.

## 6. Concluding Remarks

Amyloid-related disorders are complex pathologies involving the impairment of many biological mechanisms. Each of these diseases has unique characteristics and at the same time, they share many common and overlapping mechanisms, such as protein misfolding, aggregation and deposition, tissue degeneration, and inflammation. Moreover, the cases with the coexistence of two or more conventionally distinct pathological features are not uncommon, for example, type-2 diabetes and AD/dementia [220], or AD with synucleinopathy [221]. Currently, there is no efficient cure for the majority of amyloid-related disorders. Under physiological conditions, many intrinsic regulatory mechanisms in the body prevent protein from misfolding and abnormal aggregation. Several endogenous proteins have been found to play a key role in these mechanisms by acting as molecular chaperons with strong anti-amyloidogenic activities. Failures in the function of these proteins could become a trigger for disease initiation and/or progression. In the current review, we discussed some of these proteins that have been more extensively studied. Evaluation of available data allows us to conclude that each of these proteins is involved in the regulation and control of several amyloidogenic proteins, often by overlapping or complementary mechanisms, and most likely, their synergistic functioning helps to maintain normal protein homeostasis and protection from pathological alterations in the body. In vivo, their amyloid interfering effect should consequently be viewed as an ensemble that simultaneously can target different parts in the path from a native monomer to a mature fibril. Experimental data show that these endogenous proteins have potential therapeutic value in the treatment of amyloid-related diseases. Their endogenous production is an advantage as they are already at the correct location. However, their targeted modulation or exogenous administration are possible modes of intervention. Thus, understanding the mechanisms of action for these proteins will help to develop new therapeutic approaches that can tune the intrinsic regulatory mechanisms towards prevention of the disease development and reversion of pathological processes towards normalization.
